# Supplementary material for: Patterns of Intron Gain and Loss in Fungi
Source: PLoS Biol. 2004 Nov 30;2(12):e422. doi: 10.1371/journal.pbio.0020422 (PMC532390; doi:10.1371/journal.pbio.0020422)
Supplement: Table S1 — Also available at http://genes.mit.edu/NielsenEtAl/. (4.3 MB ZIP). [file pbio.0020422.st001.zip › NielsenEtAl/html/125.html]

AN7353.1.NCU09794.1.MG08115.1.FG02628.1


```
 CLUSTAL W (1.82) Multiple Sequence Alignments - Introns Inserted


Sequence 1: MG08115.1	350 aa
Sequence 2: FG02628.1	358 aa
Sequence 3: AN7353.1	344 aa
Sequence 4: NCU09794.1	357 aa
Alignment Length: 382 aa
Number Identitical Residues: 78 aa
Alignment Score (without introns) 4277


MG08115.1 	--------MAPIGIALIGGG~IFIK~QA~HI0PAVLATPSLEVKAVYSRSLKSAKESAAL
NCU09794.1	----------MVGIALLGAG~IFAR~EQ1HL~PAIESVPSLSLKAIYSRSESSANTLASA
FG02628.1 	MDAEWRGNETPASNTFSLKQ~LPTS1LF~PQ~PAIVKTNNLDLKAIYSRSLKSAQETAAL
AN7353.1  	---------MTIGVAIIGSG1IFAR~EQ~HL0PAIKEAPMFSLKAIYSRSLKSAQGLAEG
          	            . ::     :          **:  .  :.:**:**** .**:  *  

MG08115.1 	LPNG-----GEGVDLYSSE----EGGG-------GYADVLKRDDVSGVIIA~LPIANQPE
NCU09794.1	ASSP--------VDVYYDVPTAGSGSGEAANVEKTLDALLARTDIDAVVIA~LPILAQPA
FG02628.1 	DTRGTVAGSGSGPELYSAD----SGSGK------SLNDLLARDDIDAVIIA~LPIPSQPE
AN7353.1  	LEG---------VDLYSDD----SGAG------KSYQDLLARQDIGAVILA2LPIVAQPA
          	             ::*       .*.*      .    :* * *:..*::* ***  ** 

MG08115.1 	YIKAALDAGKHVLAEKPVAPNVSQGRELIEYYHS---IKARTGATWSCAEQMRCMPNYIF
NCU09794.1	VIEKALKAGKHVLSEKPVAKDVEEAKRLVGFYEALDLDNNKKKPLWAVAENFRYTPSLLK
FG02628.1 	YIQAALAAGKHVLAEKPIAPSVADANKLIEYYRS---LQGKG--TFSIAENFRYKPCFEY
AN7353.1  	YIKEALAAKKHVLSEKPIAKDLATAQDLMAWYND-NANVDKSKTFWGVAENFRFIRKWLK
          	 *: ** * ****:***:* .:  .. *: :*.  .    :  . :. **::*       

MG08115.1 	GAAEARKLGK-ITGFRAIVEFMTKED1NPYYN~--TEW~RKNPTHDYGFILDGGVHWAAS
NCU09794.1	AAEKVKEIGGKLTTFRLNMNGWVEEG~NKYF-~-KTEW1RKVPSYQGGFLLDGGVHFIAG
FG02628.1 	AANEAAKLGA-LQHFSVRVLFFMSED~SQWYG~--TSW~RAKPQFQGGFLLDGGVHFVAA
AN7353.1  	TAEEVQKLGG-VKTFRVVVRNKVGTE~GKYFS1IDTPW~RKIPEYQGGFLLDGGIHTVAG
          	 * :. ::*  :  *   :        . ::.  .* * *  * .: **:****:*  *.

MG08115.1 	LRMLLG--DDVAETVSGFSHTASAYLPSPDTLQGIVRTKSGASGAFLVSCGSTLPAKSEF
NCU09794.1	LRLLLSELNQEITHVAGFSALLEERLLPVDTVHAVALTNDGKSGTISISFGTQFKTGLEV
FG02628.1 	MRQLF---DEPAADVSAFTSQVQPHLAPVDTVNAVIRLKSGVSGTYQQSCG-TKMSTSAF
AN7353.1  	VRLILGRGKNSLKSLTAQTSQLQEHLPPIDTVDALLTTASGAAGVFSLSFGSEFKS-FVL
          	:* ::.  .:    ::. :   .  * . **:..:    .* :*.   * *:   :   .

MG08115.1 	SVACERGSVTVSRDNITVSRTDGSEPQVTIKNSAEGVKYEVQAWAAALEAGKPDPRLAPE
NCU09794.1	EVVTTQGSVYWNPTQIKTKTKEGEETEEFEKS--TGVKEEMAAFAKAIEKGQTEKEQTPE
FG02628.1 	QFGYEKGSVVVDGDKVTVTPWEG-EQVVKDFERTSGVDEEIEAWANALVDGKPDKRQSVE
AN7353.1  	EFTCEKGVVSLVDDQLTVN---GVATEIPFEG--VGVKEEVKAFGESIVNGELNKKLVPD
          	..   :* *     ::...   *            **. *: *:. ::  *: : .   :

MG08115.1 	EGLADAEFLLGMLKSADAGGVPQNLSLQHV
NCU09794.1	EALKDLEVVQRLLESGE--GKGIVKAVEA-
FG02628.1 	EALADLELLEMMFESGSDGGAAKKYQHQF-
AN7353.1  	EALADLEVLEMMFRSGEDGGRKTLKLQD--
          	*.* * *.:  ::.*.. .*       :
```
